# Propuesta Para la Realización de Vídeo-EEG Prolongados en Urgencias en un Hospital Secundario sin Neurólogo de Guardia

**DOI:** 10.31083/RN33466

**Published:** 2025-04-03

**Authors:** Virgilio Hernando-Requejo, Clara Horcajo-Gómez, Nuria Huertas-González

**Affiliations:** ^1^Sección de Neurología, Hospital Universitario Severo Ochoa, Leganés, 28914 Madrid, España; ^2^Hospital Universitario HM Sanchinarro, 28050 Madrid, España; ^3^Facultad de Medicina, Universidad San Pablo CEU, 28668 Madrid, España

El vídeo-electroencefalograma (VEEG) se ha consolidado como prueba complementaria fundamental en epilepsia. Su capacidad diagnóstica aumenta si se realiza pronto tras una crisis no provocada [1, 2, 3, 4]. En diversos artículos, al analizar el VEEG en las primeras 12–48 h tras la crisis en estudio se reportan anomalías en el 48,6–70% de los registros [1, 3, 4, 5, 6]. Específicamente en nuestro hospital, hemos revisado pacientes con una primera crisis no provocada y exploración neurológica, analítica y tomografía axial computarizada (TAC) craneal normales; los registros de Urgencias en las primeras 24 tras la crisis mostraron actividad epileptógena o crisis en el 63% de los pacientes (en el 26% se registraron crisis); la duración media de los estudios fue de 108.53 minutos. Gracias a estos tiempos de registro obtuvimos trazados de sueño en el 70% de los casos, y mayor capacidad para detectar anomalías, pues estas aparecieron tras 20 minutos (el estándar de un VEEG) en el 42% de los casos [5].

Por el contrario, si postponemos el VEEG y lo realizamos ambulatoriamente, se registrarán anomalías en el 12–50% [7] en un primer estudio, y si realizamos tres aumentará al 70% [8]. De modo que un VEEG en las primeras 24 h y de 100–110 minutos aporta una rentabilidad similar a tres estudios ambulatorios, pero mucho antes. Veamos los tiempos para ambos tipos de VEEG en nuestro hospital; se trata de un centro secundario de 400 camas:

Electroencephalography (EEG) ambulatorios: desde que se llama al paciente en la sala de espera para acudir a consulta hasta que el paciente la abandona con un EEG de 20 minutos: media 36 minutos, durante los cuales la consulta de enfermería está ocupada.

EEG en Urgencias: total 21 minutos, en dos tiempos: 13 para ir a Urgencias, colocar los electrodos, iniciar el registro y volver, después 100–110 minutos de registro, durante los cuales la consulta está desocupada y se puede realizar otra actividad, y luego 8 minutos para ir a urgencias, retirar los electrodos y volver a consulta.

Con estos tiempos, realizar tres EEG ambulatorios supondría 108 minutos de trabajo por paciente, mientras que, si la coordinación es adecuada (y enfermería aprovecha para otras actividades los 100–110 minutos del registro), con un estudio en Urgencias que llevaría 21 minutos obtendríamos la misma rentabilidad diagnóstica mucho antes.

El nuestro es un hospital secundario sin neurólogo de guardia, no disponemos de EEG urgente las 24 horas del día, pero lo suplimos con EEG de 100–110 minutos en las primeras 24 horas tras la crisis, por el momento 5 días por semana. El número de EEG ambulatorios ha disminuido significativamente y los tiempos de enfermería se han acortado. Consideramos que este “cambio de estrategia” en la realización de EEG podría ser beneficioso en hospitales de las características del nuestro.

## Data Availability

Data is available upon request.

## Contribuciones de los Autores

VHR: conceptualización; CHG: recogida de datos; NHG: revisión, preparación de referencias. Todos los autores contribuyeron a realizar cambios editoriales en el manuscrito. Todos los autores leyeron y aprobaron el manuscrito final. Todos los autores participaron lo suficiente en el trabajo y aceptaron ser responsables de todos los aspectos del mismo.

## Aprobación Ética y Consentimiento Informado

No aplicable.

## Agradecimientos

No aplicable.

## Financiación

Esta investigación no recibió financiación externa.
